# Correlation Between Body Mass Index and Blood Pressure Levels Among Hypertensive Patients: A Gender-Based Comparison

**DOI:** 10.7759/cureus.10974

**Published:** 2020-10-15

**Authors:** Faran Khalid, Abubakkar Siddique, Jamil Ahmed Siddiqui, Ghazala Panhwar, Simran Singh, Adnan Anwar, Atif A Hashmi

**Affiliations:** 1 Internal Medicine, Dow University Hospital, Dow University of Health Sciences, Karachi, PAK; 2 Physiology, Islamic International Medical College, Rawalpindi, PAK; 3 Biochemistry, Fazaia Ruth Pfau Medical College, Karachi, PAK; 4 Biochemistry, Al-Tibri Medical College, Karachi, PAK; 5 Biochemistry, Al-Tibri Medical College and Hospital, Karachi, PAK; 6 Internal Medicine, Jinnah Sindh Medical University, Karachi, PAK; 7 Physiology, Al-Tibri Medical College, Karachi, PAK; 8 Stereotactic Radiosurgery/Radiation Oncology, Al-Tibri Medical College, Karachi, PAK; 9 Pathology, Liaquat National Hospital and Medical College, Karachi, PAK

**Keywords:** body mass index, systolic, blood pressure, diastolic blood pressure

## Abstract

Objective

Blood pressure (BP) has been found to rise among populations due to the high body mass index (BMI). Overweight and persons who have high BP are prone to develop heart diseases. The objective of this study was to evaluate the correlation between BMI and BP among hypertensive patients in both males and females aged 18 years and above.

Methodology

A cross-sectional study was carried out among patients with a self-reported history of hypertension and anti-hypertensive medication. After taking ethical approval, a total of 337 patients aged 18 or above were selected by using convenience sampling. The duration of the study was six months. A detailed history was taken from each patient about hypertension associated symptoms with the help of a self-designed questionnaire. The BMI of the patients was assessed. Statistical Package for Social Sciences (SPSS) Version 20.0 (IBM Corp., Armonk, NY, USA) was used to analyze the collected data. Spearman correlation was used, and p-value <0.05 was considered significant.

Results

In a total of 337 patients, the mean age of the patients was 45.87±13.38 years. In which 176 (52.2%) were males and 161 (47.8%) were females. Their mean BMI level was 26.83±5.83 kg/m^2^, and the mean systolic blood pressure level was 141.78±13.00 mm Hg whereas the diastolic blood pressure was 85.21±10.03 mm Hg. The results also showed that among males the BMI had a significant negative correlation with both systolic blood pressure level (ρ = -0.212, p = 0.011) and diastolic blood pressure level (ρ = -0.208, p = 0.013), while in females the correlation was insignificant.

Conclusion

Our study results concluded that the BMI of the patients had a significant weak negative correlation with both systolic blood pressure level and diastolic blood pressure level in males; however, no significant correlation was found in females.

## Introduction

Presently, the occurrence of obesity in European countries ranges from 10% to 20% for men and 10% to 25% for women [1]. A report from the Centers for Disease Control and Prevention in 2002 revealed that the overweight rate was 65%, and the obesity rate was 31% [2]. In a study on the epidemiology of obesity, the incidence of obesity was found to be higher among females as compared to males [3]. The increasing rate of overweight and obesity in the developing countries contributed to the rapid incline in nutritional, epidemiologic, and socio-economic burden [4].

As stated by the World Health Organization (WHO), overweight and obesity have become so common that they are substituting the more traditional public health distresses, such as undernutrition and contagious diseases [5]. Worldwide, the incidence of high blood pressure (HBP) in adults aged 25 and over was around 40% in 2008. According to WHO, the prevalence of high blood pressure was highest in Africa (46%) for both sexes [6]. In 2003, the occurrence of hypertension in urban areas in Ghana was found to be 28.3% in the Greater Accra Region [7] and 28.7% in the Ashanti Region [8]. Body mass index (BMI) is a scale to measure human body weight in connection with the height, calculated by dividing the weight in kg by the square of the height in meters [9]. WHO has categorizes BMI as normal (18.5-25 kg/m^2^), overweight (26-30 kg/m^2^), and obese (>30 kg/m^2^) [10].

Research in Pakistan has shown that around 30% of cases of hypertension may be due to obesity in men under the age of 45 years. However, in some cases, this figure may be as high as 60% [11]. Another study in Pakistan revealed that overweight and obesity in college students is almost 40%, which is very alarming [12]. Obesity is the main challenge for Pakistani people because we are inherently prone to it. Blood pressure (BP) is the resistance of blood flow against the walls of the arteries [13]. Systolic blood pressure (SBP) is the highest level, which refers to the amount of pressure in our arteries during contraction of our heart muscle. Diastolic pressure, however, is the pressure obligatory to allow continuous flow in the blood vessels and filling of the ventricles before the next systole [14]. Many other studies have revealed that BMI and BP are both increasing worldwide, and epidemiological studies showed a positive correlation between both of them [15]. One study described that the causes of overweight and obesity are complicated [16]. Some aspects seem to play a significant part, like consuming too many calories and lack of physical exercise. In the last few years, the growing trend of taking junk food, lack of physical activity, especially in the younger generation, and sedentary activities are liable for the rising incidence of non-communicable diseases like diabetes mellitus (DM), hypertension (HTN), ischemic heart disease (IHD) and obesity [17].

The objective of this study was to evaluate the correlation between BMI and BP amongst hypertensive patients in both males and females aged 18 years and above.

## Materials and methods

This cross-sectional study was carried out among patients with a self-reported history of hypertension and anti-hypertensive medication. After taking ethical approval, data were collected from the medical outpatient department of a secondary care hospital in Karachi. A total of 337 patients aged 18 or above were included in the study by using convenience sampling. The duration of the study was six months from January 2019 till July 2019.

A detailed history was taken from each patient about hypertension-associated symptoms with the help of a questionnaire designed specifically for the study. Patients with a history of diabetes, cardiac events, neurological disorders, cluster headache, gastrointestinal disease, and morbid obesity were excluded from the study. Age and gender were recorded. Blood pressure was measured using a sphygmomanometer with a stethoscope, and BMI was classified according to WHO classification [10].

Data were analyzed using Statistical Package for Social Sciences Version 20.0 (IBM Corp., Armonk, NY, USA). The descriptive analysis was performed by calculating means and standard deviations, whereas qualitative data were expressed as frequency and percentages. Spearman correlation was assessed. P-value <0.05 was set as statistical significance.

## Results

In a total of 337 patients, the study results showed that the mean age of the patients was 45.87±13.38 years; 176 (52.2%) of them were males while 161 (47.8%) of them were females. Their mean BMI level was 26.83±5.83 kg/m^2^. The mean systolic blood pressure level was 141.78±13.00 mm Hg, whereas the mean diastolic blood pressure level was 85.21±10.03 mm Hg (Table 1).

**Table 1:** General characteristics of patients (n = 337) SD, standard deviation; BMI, body mass index

Variables (n=337)	Mean±SD/Count (%)
Age (years)	45.87±13.38
Gender	
Male	176(52.2)
Female	161(47.8)
BMI (kg/m^2^)	26.83±5.83
Systolic blood pressure (mm Hg)	141.78±13.00
Diastolic blood pressure (mm Hg)	85.21±10.03

The study results further revealed that the BMI of the patients had a significant weak negative correlation with both systolic blood pressure level (ρ = -0.137, p = 0.024) and diastolic blood pressure level (ρ = -0.126, p = 0.039) of the patients (Table 2).

**Table 2:** Correlation between BMI and systolic and diastolic blood pressure BMI, body mass index

	Variable	Systolic Blood Pressure		Diastolic Blood Pressure	
		ρ	p	ρ	p
	Overall	-0.137	0.024	-0.126	0.039
BMI (kg/m^2^)	Male	-0.212	0.011	-0.208	0.013
	Female	-0.065	0.465	-0.063	0.477

The study results also showed that among males, the BMI of the patients had a weak significant negative correlation with both systolic blood pressure level (ρ = -0.212, p = 0.011) and diastolic blood pressure level (ρ = -0.208, p = 0.013) of the patients, while among females, the BMI of the patients had no significant correlation with either systolic blood pressure level (ρ = -0.065, p = 0.465) or diastolic blood pressure level (ρ = -0.063, p = 0.477) of the patients (Table 2).

## Discussion

In the National Health Survey of Pakistan (NHSP), the overweight prevalence was 13.5% for males and 19.6% for females, whereas the underweight population was 25%, which is not comparable with our study. According to NHSP, the mean BMI was 21.3kg/m2 for males and females, while in our study, the mean BMI in males and females was 26.83kg/m^2^, which is slightly higher than NHSP [18]. Another study conducted on Pakistani population showed the prevalence of obesity, overweight and underweight was 2.2%, 14%, and 11%, respectively. In a study conducted by Shams et al. on female medical students, there were 47 (15%) underweight, 62 (20%) overweight, and 78 (25.4%) obese female students [12]. Changes in lifestyle and eating behavior in youngsters in the past are the key factors for the difference in obesity values in studies. A study by Bertsias et al. showed the prevalence of central obesity as 13% [19].

Another study reported that age is also a main factor that affects BMI. Throughout the age groups, the highest rate of obesity (4.0%) was documented by the 46-50 years’ age group in their research. The increased rate of obesity in people with an increase in age was due to the reduced physical activity in spite of retaining the same energy intake in their past years. Therefore, the surplus energy that is not used is stored as fat, thus increasing the risks of becoming obese [20]. People are becoming less physically active due to growth. Nowadays, a number of people wish to use cars even to go within walking distance. This study found that only 37 (18.5%) of the members exercised [21]. Hence, it is not surprising that there is a high rate of overweight and obesity since a reduction in physical activity (sedentary lifestyle) is linked with overweight and obesity [21].

We found a weak negative correlation between BMI and systolic (ρ = -0.212, p = 0.011) and diastolic blood pressures (ρ = -0.208, p = 0.013) in males (Table 2). The correlation between BMI and systolic and diastolic blood pressures in females, the BMI of the patient was statistically insignificant (ρ = -0.065, p = 0.465) and (ρ = -0.063, p = 0.477), respectively (Table 2).

In our research, there is an absence of consistency with other studies as the overall results of our study revealed and that the BMI of the patients had a significant weak negative correlation with both systolic blood pressure level (ρ = -0.137, p=0.024) and diastolic blood pressure level (ρ = -0.126, p=0.039) of the patients (Table 2). The link between these two pressures with BMI has been well presented in various studies, along with the risk factors of cardiovascular diseases [22]. The importance of prevention, as well as control of weight and obesity, is almost constant in our study results. These findings also emphasize that maintaining and measuring blood pressure and body weight and timely diagnosis and control are essential for overweight and obese people. However, our study might not be immune to observer and selection bias. Considering the observations of our study and the fact that BMI correlates with lifestyle, it will be interesting to discover more facts regarding the correlation of lifestyle with blood pressure.

## Conclusions

The correlation analysis of our study showed a significant weak negative correlation of BMI with both systolic and diastolic blood pressure levels for male patients. On the other hand, no significant correlation was noted in females. Therefore, it can be concluded that, due to the weak negative correlation, the BMI does not affect blood pressure.
